# Heterogeneous Responses to Mechanical Force of Prostate Cancer Cells Inducing Different Metastasis Patterns

**DOI:** 10.1002/advs.201903583

**Published:** 2020-06-17

**Authors:** Zhixiao Liu, Liujun Wang, Huan Xu, Qiqige Du, Li Li, Ling Wang, En Song Zhang, Guosong Chen, Yue Wang

**Affiliations:** ^1^ Research Center of Developmental Biology Department of Histology and Embryology College of Basic Medicine Second Military Medical University Shanghai 200433 China; ^2^ Department of Urology Shanghai General Hospital Shanghai Jiao Tong University School of Medicine Shanghai 201620 China; ^3^ Department of Urology Changhai Hospital of Shanghai Second Military Medical University Shanghai 200433 China; ^4^ Department of Pathology Duke University School of Medicine Durham NC 27710 USA; ^5^ The State Key Laboratory of Molecular Engineering of Polymers and Department of Macromolecular Science Fudan University Shanghai 200433 China; ^6^ Stem Cell and Regeneration Medicine Institute Research Center of Translational Medicine Second Military Medical University Shanghai 200433 China; ^7^ Shanghai Key Laboratory of Cell Engineering Second Military Medical University Shanghai 200433 China

**Keywords:** extracellular microenvironment, mechanical force response heterogeneity, physical cues, prostate cancer metastasis

## Abstract

The physical cues in the extracellular environment play important roles in cancer cell metastasis. However, how metastatic cancer cells respond to the diverse mechanical environments of metastatic sites is not fully understood. Here, substrates with different mechanical properties are prepared to simulate the extracellular mechanical environment of various human tissues. The prostate cancer (PC) cells derived from different cancer metastasis sites show heterogeneity in mechanical response. This heterogeneity mediates two distinct metastasis patterns. High stiffness promotes individual cell migration and proliferation by inducing Yes‐associated protein and tafazzin (YAP/TAZ) nuclear localization in bone metastasis‐derived cells, whereas low stiffness promotes cell migration and proliferation by inducing lymphatic metastasis‐derived cells to form clusters characterized by high expression of CD44. The different metastasis patterns induced by the mechanical properties of the extracellular environment are crucial in the development of PC.

## Graphical abstract

Cancer metastasis is essential for cancer deaths. The cancer cells are regulated by the mechanical properties of the metastatic site. The prostate cancer cells derived from different cancer metastasis sites show heterogeneity in mechanical response. The different metastasis patterns induced by the mechanical properties of the extracellular environment are crucial in the development of prostate cancer.

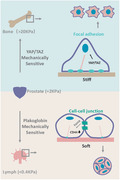

## 1 Introduction

Cancer metastasis is essential for cancer deaths.^[^ ^1^ ^]^ Prior studies have shown that the organ‐specific metastasis of cancer cells is due to selective survival on the extracellular microenvironment.^[^ ^2^ ^]^ The influence of particular biochemical microenvironmental factors upon cancer metastasis had been intensely studied.^[^ ^3^ ^]^ However, little is known about the dependence of metastatic cancer cells on the specific mechanical properties of metastatic sites.

**Figure 1:**
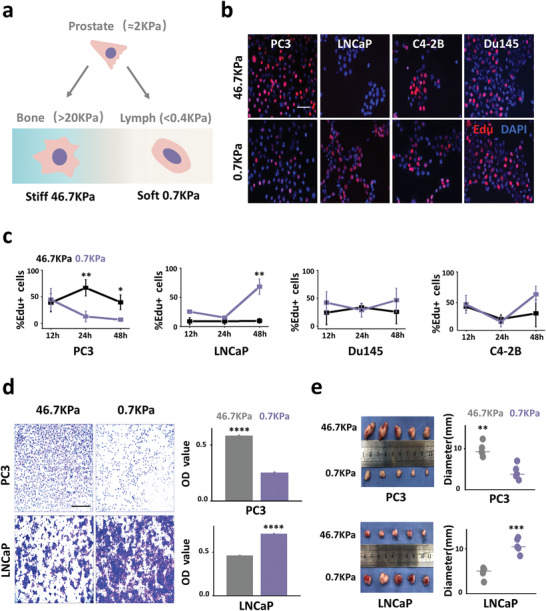
Prostate cancer cells from different metastatic sites show heterogeneous responses to mechanical force. a) Prostate cancer metastasis varies according to mechanical force. To build an ideal model for assessing the mechanical response characteristics of cancer cells at different metastatic sites, substrates differing in stiffness were engineered to simulate tissues of varying mechanical strength. b) EdU staining results (scale bar, 100 µm). Prostate cancer cells from various metastasis sites were plated on substrates with different mechanical properties (stiffness values of 0.7–46.7 KPa), and EdU staining was used to detect cell proliferation after 48 h of culture. c) Fluorescence immunoassay analysis of cell proliferation under different culture times (12, 24, and 48 h, *n* = 3). PC3 cells grown on stiff substrates (46.7 KPa) have a higher proliferation rate than PC3 cells grown on soft substrates (0.7 KPa) at 24 and 48 h (*p* = 0.0034 and *p* = 0.0167, respectively). In contrast, LNCaP cells grown on soft substrates have a higher proliferation rate at 48 h (*n* = 3, *p* = 0.0025). There was no significant difference in the proliferation of Du145 and C4‐2B cells cultured on different substrates. d) Transwell crystal violet staining results (scale bar: 1 mm). PC3 and LNCaP cells were cultured for 72 h on different substrates and cell migration was evaluated using Transwell assays. The crystal violet was eluted from cells with acetic acid, and absorbance was measured at 570 nm to determine the number of migrated cells. PC3 cells grown on stiff substrates have a higher migration efficiency (*n* = 3, *p* = 1.4297 × 10^−10^), whereas LNCaP cells grown on soft substrates have a higher migration efficiency (*n* = 3, *p* = 3.8044 × 10^−7^). e) In vivo tumorigenic ability of PC3 and LNCaP cells after 7 days of culture on different substrates. PC3 cells grown on stiff substrate have a larger diameter than those grown on soft substrates (*n* = 5, *p* = 0.0014), whereas LNCaP cells grown on soft substrate form larger diameter tumors (*n* = 5, *p* = 0.0003). *p<0.05; ** *p<*0.01; *** *p<*0.001; **** *p<*0.0001, *t*‐test.

Every tissue in the human body has its specific mechanical strength and the difference is enormous. For example, the modulus of the brain tissue or body fluid system is only 0.2 KPa, and the modulus of the bone is higher than 20 KPa or even 4 GPa can be achieved.^[^ ^4^ ^]^ These tissues with different mechanical strengths provide extremely heterogeneous extracellular mechanical environments for metastatic cancer cells. Studies demonstrated that extracellular environment mechanical signals are involved in metastasis including proliferation, epithelial‐mesenchymal transition (EMT) of cells, tumor stem cell formation, and glycolysis process.^[^ ^5^, ^6^, ^7^, ^8^, ^9^, ^10^ ^]^ However, how metastatic cancer cells shed from the primary homogeneous mechanical environment adapts to metastatic sites with heterogeneous mechanical properties has not yet been discussed. Therefore, we prepared substrates with different stiffness values (from 46.7 to 0.7 KPa) to simulate the mechanical strength of tissues.^[^ ^11^, ^12^ ^]^ Substrates with different mechanical properties were used to study the response of cancer cells from different metastatic sites to external environmental mechanical forces. Prostate cancer (PC) cell lines generated from different metastatic tissues were selected for study for the difference in mechanical strength between these tissues was significant (**Figure** **1**a).^[^ ^13^, ^14^ ^]^

Our results show that cells from different metastatic sites are heterogeneous in response to external environmental mechanical forces, and mediate two different metastatic patterns. High stiffness substrate promotes migration and proliferation of bone metastases‐derived cells by inducing YAP/TAZ nuclear localization. Low stiffness substrate promotes migration and proliferation of lymphatic metastasis‐derived cells by inducing cells to form CD44 high expression clusters. This mechanical response heterogeneity indicates that cancer cells adapt to the mechanical properties of the metastatic site by adjusting their mechanical response pattern.

## 2 Results

### 2.1 PC Cells Metastasis Sites Diversity Contains Mechanical Force Response Heterogeneity

Four different PC cell lines (PC3, bone metastasis; LNCaP, lymphatic metastasis; Du145, brain metastasis; and C4‐2B, bone metastasis cells derived from LNCaP) were used to study the responses to mechanical force of cancer cells from different metastatic sites. According to previous studies,^[^ ^12^ ^]^ we used polydimethylsiloxane (PDMS) to prepare substrates with different stiffness values (0.7–46.7 KPa), and Sulfo‐SANPAH to crosslink collagen‐I on the PDMS surface (Figure S1, Supporting Information) for collagen is involved in the development of PC.^[^ ^15^, ^16^ ^]^ Then, cells were plated on substrates differing in stiffness, and proliferation was measured using 5‐ethynyl‐2′‐deoxyuridine (EdU) staining. The results showed that different cell lines have heterogeneous mechanical responses to substrates varying in stiffness. Bone metastasis‐derived PC3 cells proliferated faster on 46.7 KPa substrate, whereas lymphatic metastasis‐derived LNCaP cells proliferated faster on 0.7 KPa substrate. There was no significant difference between the proliferation of Du145 and C4‐2B cells on the two substrates (Figures 1b,c). To further investigate the behavioral differences between PC3 and LNCaP cells on different substrates, we assessed cell migration using Transwell assays. PC3 cells cultured on 46.7 KPa substrate had higher migration efficiency, whereas LNCaP migration was more efficient on 0.7 KPa substrate (Figure 1d).

Previous studies showed that the regulation of cellular behavior by external environmental forces is irreversible.^[^ ^17^ ^]^ Our results also show that external environmental forces irreversibly regulate the behavior of PC cells (Figure S2a,b, Supporting Information). Therefore, after 7 days of culture on substrates differing in stiffness, PC3 and LNCaP cells were collected and injected subcutaneously into nude mice to simulate tumors^[^ ^18^ ^]^ and investigate whether the regulation of cell behavior by mechanical forces can be maintained in vivo (Figure S2c, Supporting Information). The average diameter of tumors formed by PC3 cells grown on 46.7 KPa substrate was larger compared to those grown on 0.7 KPa substrate. In contrast, LNCaP cells grown on 0.7 KPa substrates formed larger diameter tumors (Figure 1e).

### 2.2 YAP/TAZ Nuclear Localization Mediates Stiff Environment Induced Cancer Metastasis

**Figure 2:**
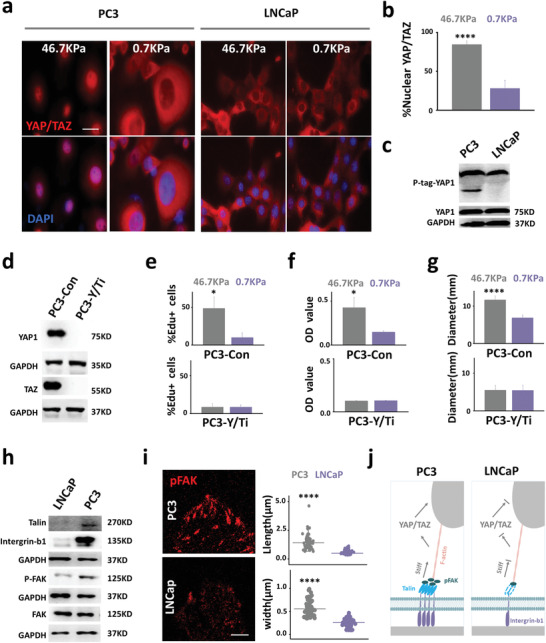
YAP/TAZ nuclear localization mediates stiff environment‐induced cancer proliferation and metastasis. a) Immunofluorescence imaging of YAP/TAZ in cells grown on different substrates (scale bar, 20 µm, 48 h). Stiff substrate regulation of YAP/TAZ nuclear localization is heterogeneous in cell lines derived from different metastatic sites. Stiff substrate induces YAP nuclear localization in PC3 cells, but not in LNCaP cells. b) Higher YAP/TAZ nuclear localization ratio was observed in PC3 cells grown on stiff substrates compared with PC3 cells grown on soft substrates (*n* = 3, *p* = 8.1573 × 10^−6^). c) Phos‐Tag sodium dodecyl sulfate‐polyacrylamide gel electrophoresis (SDS‐PAGE) (P‐tag) demonstrates YAP1 dephosphorylation levels in PC3 and LNCaP cells grown on stiff substrates (46.7 KPa). The stiff substrate induces YAP1 dephosphorylation failure in LNCaP cells. d) TAZ and YAP expression in cells transfected with empty vector (PC3‐Con) and YAP1/TAZ‐interfering RNA (PC3‐Y/Ti). e) The proliferative capacity of PC3‐Con cells and PC3‐Y/Ti cells grown on different substrates was evaluated by EdU staining (*n* = 3, *p* = 0.0149). f) The migration ability of PC3‐Con and PC3‐Y/Ti cells growing on different substrates was assessed using Transwell assays (*n* = 3, *p* = 0.0160). g) Tumor diameter in nude mice. PC3‐Con cells and PC3‐Y/Ti cells were grown on different substrates for 7 days and injected into subcutaneous tumors in nude mice; the tumor diameter was measured 4 weeks after injection (*n* = 5, *P* = 5.6823 × 10^−5^). h) The expression of the YAP1 mechanical induction nuclear localization‐related proteins talin, integrin‐b1, and p‐FAK (phosphorylated FAK [Tyr397]) in PC3 and LNCaP cells. i) Immunofluorescence imaging of pFAK in PC3 and LNCaP cells (scale bar, 2.5 µm). The difference in focal adhesion morphology between PC3 and LNCaP cells was revealed by p‐FAK; PC3 cells had longer (*n* = 53, *p* = 1.6069 × 10^−13^) and wider (*n* = 53, *p* = 1.7690 × 10^−23^) focal adhesions than LNCaP. j) Schematic showing that the response heterogeneity of different metastasis site‐derived cells to stiff substrates is due to differences in YAP/TAZ localization. *p<0.05; ** *p<*0.01; *** *p<*0.001; **** *p<*0.0001, *t*‐tests.

The YAP/TAZ localization of cells grown on different substrates was analyzed, since YAP/TAZ mediates the cellular response to stiff substrates. Previous studies have shown that stiff substrates induce YAP dephosphorylation and cause YAP nuclear localization to promote cell proliferation.^[^ ^13^, ^19^ ^]^ Stiff substrates promoted greater nuclear localization of YAP/TAZ in PC3 cells compared to soft substrates (**Figures** **2**a,b). In cells that do not respond to stiff substrates, such as LNCaP, Du145, and C4‐2B, stiff substrates failed to induce nuclear localization of YAP/TAZ (Figure 2a and Figure S3a–c, Supporting Information). Phos‐Tag sodium dodecyl sulfate‐polyacrylamide gel electrophoresis (SDS‐PAGE) was used to detect YAP1 phosphorylation levels; YAP/TAZ was activated and localized to the nucleus after dephosphorylation^[^ ^19^, ^20^ ^]^ and YAP1 was used to represent YAP and TAZ.^[^ ^19^ ^]^ In PC3 but not LNCaP cells, YAP1 was dephosphorylated on a stiff substrate (Figure 2c). Stiff substrates also failed to induce YAP1 dephosphorylation in other cells that were nonresponsive to stiff substrates (Figure S3d, Supporting Information).

YAP/TAZ localization is affected by cell area.^[^ ^19^ ^]^ There was no significant change in the morphology of PC3 cells grown on substrates with various mechanical properties (Figure S4a, Supporting Information), as reported previously.^[^ ^12^ ^]^ Next, stiff substrate‐induced YAP/TAZ nuclear localization in PC3 cells was discussed. Previous studies have shown that stiff substrate mechanical signals are transmitted into cells through the following pathways: focal adhesion→ pFAK→ cytoskeleton tension and eventually induce YAP/TAZ dephosphorylation. F‐actin is involved in this process.^[^ ^19^, ^21^ ^]^ Cells were treated with inhibitors of focal adhesion kinase (FAK) (PF‐573228),^[^ ^22^ ^]^ F‐actin (cytochalasin B),^[^ ^23^ ^]^ and cytoskeleton tension (Y‐27632).^[^ ^19^ ^]^ YAP/TAZ nuclear localization in PC3 cells decreased after incubation with the inhibitors for 3 h, and the difference in PC3 localization among substrates differing in stiffness was no longer significant (Figure S4b,c, Supporting Information). Phos‐Tag SDS‐PAGE analysis further demonstrated that the inhibitors prevented dephosphorylation of YAP1 (Figure S4d, Supporting Information). Interestingly, soft substrates did not inhibit dephosphorylation of YAP1 in PC3 cells, which may be because the lack of pLC*γ*1 in PC3 cells prevented the accumulation of PtdIns(4,5)P_2_ on the cell membrane (Figure S4e, Supporting Information).^[^ ^24^ ^]^ These results indicate that the nuclear localization of YAP/TAZ in PC3 cells grown on a stiff substrate is induced by the mechanical properties of the substrate.

To verify that YAP/TAZ mediates the proliferation and migration of PC3 cells on stiff substrate, YAP/TAZ expression was knocked down using RNA interference (Figure 2d). PC3 cells were transfected with an empty vector (PC3‐Con) or YAP/TAZ‐interfering RNA vector (PC3‐Y/Ti)^[^ ^19^ ^]^ and cultured separately on substrates differing in stiffness to analyze the effect of the mechanical properties of the substrate on cell behavior. When YAP/TAZ expression was knocked down, the stiff substrate no longer promoted PC3 cell proliferation (Figure 2e and Figure S5a, Supporting Information) or migration (Figure 2f and Figure S5b, Supporting Information). PC3‐Con and PC‐Y/Ti were cultured on different stiff substrates and injected subcutaneously into nude mice to simulate tumors. PC3 cells lost responsiveness to stiff substrates after YAP/TAZ expression was knocked down in vivo (Figure 2g and Figure S5c, Supporting Information).

To further explain the lack of stiff substrate‐induced YAP nuclear localization in LNCaP cells, we examined the expression of talin, integrin‐b1, and the phosphorylation of FAK, which is essential for mechanically induced YAP/TAZ dephosphorylation.^[^ ^21^ ^]^ The expression of talin, integrin‐b1, and the phosphorylation of FAK was lower in LNCaP than PC3 cells (Figure 2h). Immunofluorescence imaging showed that LNCaP cells had a smaller adhesion area than PC3 cells (Figure 2i and Figure S6, Supporting Information), which also implied negative in the stiff regulation of YAP/TAZ nuclear localization.^[^ ^21^ ^]^

A stiff substrate promotes the proliferation and migration of PC3 cells by inducing nuclear localization of YAP/TAZ. Because LNCaP cells lack mechanically activated YAP/TAZ‐related proteins, stiff substrates fail to induce YAP/TAZ nuclear localization, and therefore cannot promote the proliferation or migration of LNCaP cells (Figure 2j). The above results suggest that YAP/TAZ nuclear localization mediates the ability of stiff substrate to promote PC cell metastasis.

### 2.3 Soft Environment Promotes Cancer Metastasis by Inducing Cell Clustering

**Figure 3:**
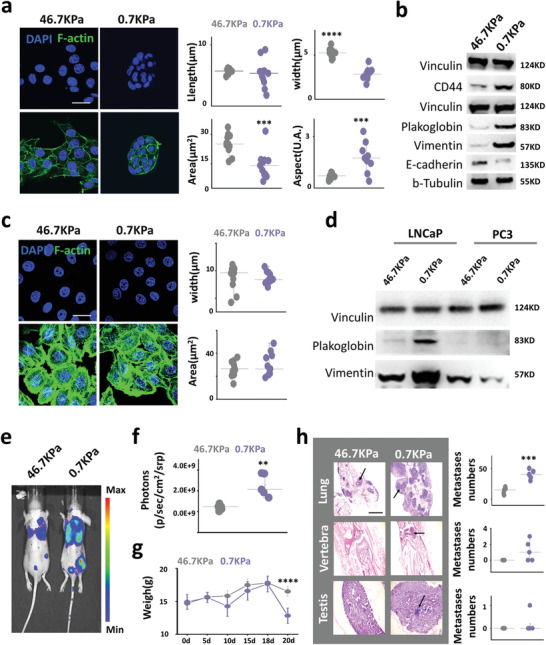
Soft substrates induce cells to form clusters and thus promote cancer proliferation and metastasis. a) F‐actin immunofluorescence imaging of LNCaP cells grown on different substrates (scale bar, 10 µm). After 4 days of culture, LNCaP cells grown on soft substrates (0.7 KPa) formed spherical clusters with thinner nuclei, smaller widths (*n* = 11, *p* = 9.5546 × 10^−9^), a larger aspect ratio (*n* = 11, *p* = 0.0003), and smaller area (*n* = 11, *p* = 0.0005) compared with LNCaP cells grown on stiff substrates (46.7KPa). All nuclei in one cluster are displayed; ten clusters were counted and showed the same characteristics. b) Circulating tumor cell (CTC) cluster ‐related (plakoglobin and CD44) and EMT‐related (vimentin, E‐cadherin) protein expression in LNCaP cells after 4 days of growth on different substrates. c) F‐actin immunofluorescence imaging of PC3 cells grown on different substrates. PC3 cells did not form spherical clusters after 4 days of culture on soft substrate, and the morphology of nuclei on cells grown on different substrates did not change significantly. d) Expression of plakoglobin and vimentin in LNCaP and PC3 cells grown on different substrates after 4 days cultured. e) Luciferase‐labeled LNCaP cells were injected into nude mice through the tail vein after 7 days of culture on different substrates and analyzed in vivo imaging after 20 days (*n* = 5). f) Image analysis showing that LNCaP cells grown on soft substrates have higher photon counts in the lungs (*n* = 5, *p* = 0.0027). g) Mice injected with LNCaP cells grown on soft substrate showed a significant decrease in body weight on day 20 after injection compared with mice injected with LNCaP cells grown on stiff substrate (*n* = 5, *p* = 2.4359 × 10^−5^). h) Hematoxylin and eosin (H&E) staining imaging of tissue sections from mice injected with LNCaP cells grown on different substrates through the tail vein (sections are from the lungs, sixth lumbar vertebra, and testicles; scale bar, 1 mm). LNCaP cells grown on soft substrates had a larger metastatic area and more metastases in the lungs compared with those grown on stiff substrates (*p* = 2.68489 × 10^−4^). Further, LNCaP cells grown on soft substrate metastasized to the vertebrae and testes, whereas those grown on stiff substrate did not (*n* = 5). *p<0.05; ** *p<*0.01; *** *p<*0.001; **** *p<*0.0001, *t*‐tests.

After 4 days of incubation, LNCaP cells formed spherical clusters on soft substrates but exhibited spread on stiff substrates (**Figure** **3**a). Next, we described the clusters induced by soft substrates in terms of nuclear morphological changes, since cell morphology was difficult to measure due to the sphere formation. After forming globular clusters, the nuclei of LNCaP cells became thinner and longer, with a smaller area in 2D imaging (Figure 3a). This nuclear morphological change, regulated by soft substrates, required intercellular contact (Figure S7, Supporting Information). The morphology of the LNCaP cluster induced by soft substrates was very similar to that of circulating tumor cell (CTC) clusters;^[^ ^25^, ^26^ ^]^ therefore, we examined CTC cluster‐associated proteins in LNCaP cells grown on substrates having different mechanical properties. Soft substrates increased plakoglobin and CD44 expression in LNCaP cells, which play a fundamental role in the formation of CTC clusters.^[^ ^25^ ^]^ Soft substrates also induced an increase in the expression of vimentin and reduced E‐cadherin expression, thereby inducing EMT in LNCaP cells (Figure 3b).^[^ ^27^ ^]^ Soft substrate does not induce N‐cadherin expression (Figure S8b, Supporting Information). However, soft substrates did not induce cells to form clusters in PC3, C4‐2B, DU145, and BPH‐1 cells (Figure 3c and Figure S8a, Supporting Information). Further, soft substrates did not promote the expression of plakoglobin and vimentin in PC3, C4‐2B, DU145, and BPH‐1 cells, even reduced the expression of vimentin in PC3 cells (Figure 3d and Figure S8c, Supporting Information). Previous studies demonstrated that CTC clusters have high metastatic potential.^[^ ^28^ ^]^ Therefore, after 7 days of culture on different mechanical substrates, luciferase‐labeled LNCaP cells were injected into nude mice through the tail vein to observe the effects of different mechanical properties on LNCaP migration ability in vivo. The spherical clusters induced by soft substrates were digested into single cells for counting, and the results showed that LNCaP cells grown on a soft substrate had higher metastatic efficiency (Figures 3e,f) and caused the nude mice to lose more weight than those cultured on a stiff substrate (Figure 3g). This trend was further confirmed by hematoxylin and eosin (H&E) staining of tissue sections (Figure 3h).

**Figure 4:**
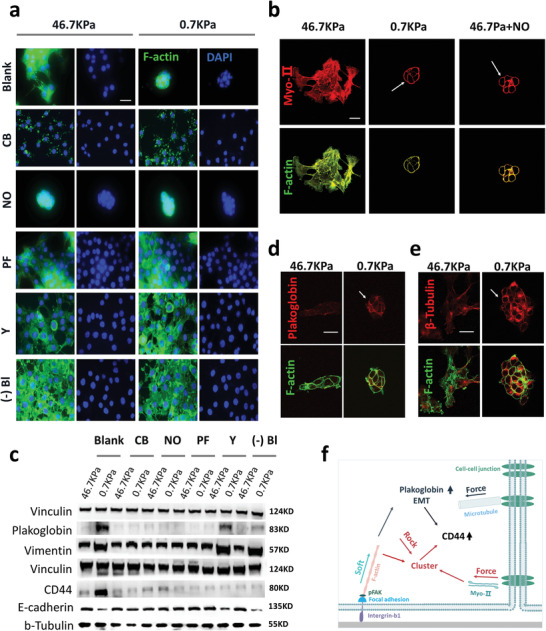
A soft substrate regulates the intracellular mechanical force response network to promote the formation of high metastatic cell clusters. a) Fluorescence imaging of LNCaP cells treated with the F‐actin inhibitor cytoskeleton B (CB), microtubule inhibitor nocodazole (NO), FAK inhibitor PF‐573288 (PF), ROCK inhibitor Y‐27632 (Y), and myosin II (Myo‐II) inhibitor (‐)‐blebbistatin ((‐)Bl). After the cells were cultured on different substrates for 3 days, inhibitors were added and incubated for 24 h. (green, F‐actin; scale bar, 25 µm). b) Immunofluorescence imaging of LNCaP cells grown on different substrates, including stiff substrates with NO (red, Myo‐II; green, F‐actin; scale bar, 10 µm, 4 days or cultured on different substrates, 3 days and co‐cultivation with NO 24 h for 46.7 Pa+NO). c) Expression of CTC cluster‐related (plakoglobin and CD44) and EMT‐related (vimentin and E‐cadherin, 4 days) proteins in LNCaP cells grown on different substrates after treatment with different inhibitors (CB, NO, PF, Y, and (‐)Bl). d,e) Immunofluorescence imaging of LNCaP cells grown on different substrates after 4 days cultured. In (d) red is plakoglobin and green is F‐actin. In (e), red is *β*‐tubulin and green is F‐actin (scale bar, 10 µm). f) Schematic diagram of the mechanism through which a soft substrate regulates the ability of LNCaP cells to form high transfer clusters. Soft substrates introduce information into cells via F‐actin, FAK, cytoskeletal tension, and Myo‐II to induce cell clustering. Soft substrates also increase the expression of plakoglobin and induce EMT via F‐actin, FAK, and microtubules. Increased clustering and expression of plakoglobin and EMT are essential for increasing the expression of CD44.

F‐actin (cytochalasin), microtubules (nocodazole),^[^ ^19^ ^]^ FAK (PF‐573228), and cytoskeleton tension (Y‐27632) inhibitors were used to discuss which mechanical components are involved in the response of the soft substrate in LNCaP cells. F‐actin, FAK, and cytoskeleton tension inhibitors inhibited soft substrate‐induced LNCaP spherical cluster formation (**Figure** **4**a). Interestingly, microtubule inhibitors stimulated LNCaP cells to form spheres, even on stiff substrates (Figure 4a and Figure S9, Supporting Information). Previous studies showed that myosin II (Myo‐II) plays an important role in mechanical conduction at cell–cell junctions and disruption of microtubules promotes the assembly of Myo‐II into filaments.^[^ ^29^, ^30^ ^]^ Therefore, we hypothesized that Myo‐II plays an important role in soft substrate‐induced clustering of LNCaP cells, and that a microtubule inhibitor would promote LNCaP clustering by stimulating Myo‐II assembly into microfilaments. Fluorescence immunoassays showed that culture on a soft substrate induced Myo‐II enrichment around the cell cluster, and the same phenomenon occurred in cells treated with microtubule inhibitors (Figure 4b). Moreover, the Myo‐II inhibitor (‐)‐blebbistatin^[^ ^31^ ^]^ inhibited LNCaP cluster formation on soft substrates (Figure 4a). In addition, western blotting showed that all inhibitors reduced soft substrate‐induced CD44 expression. Cytoskeleton tension and Myo‐II had no effect on the soft substrate‐induced increase in plakoglobin and EMT (Figure 4c).

The above results indicated that cell clustering and plakoglobin upregulation are crucial for the formation of soft substrate‐induced CD44‐expressing spherical clusters. Clustering was related to F‐actin, FAK, cytoskeletal tension, and Myo‐II. High levels of plakoglobin and EMT were associated with F‐actin, FAK, and microtubules.

To verify that the increase in CD44 expression is vital to the ability of soft substrates to promote LNCaP proliferation, we evaluated the effects of inhibitors on cell proliferation in vivo. We selected a microtubule inhibitor that inhibits plakoglobin expression but promotes cell clustering, and a Myo‐II inhibitor that inhibits cell clustering but not increase plakoglobin expression. There was no significant difference in tumorigenicity in vivo after treatment of cells grown on substrates with different mechanical properties with microtubule versus Myo‐II inhibitors (Figure S10c, Supporting Information).

Previous studies demonstrated that CTC cluster formation is accompanied by EMT,^[^ ^25^ ^]^ and that EMT also occurs during soft substrate‐induced LNCaP clustering. F‐actin, FAK, and microtubules participate in soft substrate‐induced EMT. Conversely, cytoskeletal tension and Myo‐II were not involved in soft substrate‐induced EMT. Moreover, inhibitors of these markers did not affect soft substrate‐induced enhanced cell migration ability in vitro (Figure 4c and Figure S11a,b, Supporting Information) but did inhibit soft substrate‐promoted cancer metastasis in vivo (Figure S10d, Supporting Information).

The above results suggest that soft substrates induce the collective migration of LNCaP cells. During collective migration, cell contact and empty edges are important, suggesting that cell density affects the ability of soft substrates to regulate LNCaP cell migration.^[^ ^32^ ^]^ The current results confirm that intercellular contact among LNCaP cells is necessary for soft substrate‐induced LNCaP clustering (Figure S7a and c, Supporting Information). When we assessed the behavior of high‐density LNCaP cells according to the mechanical properties of the substrate, LNCaP cells lacking empty edges due to a high cell density did not form spherical clusters, and substrate did not induce high expression of CD44, plakoglobin and vimentin (Figure S11a,b, Supporting Information).

During collective migration, the transfer of mechanical force at the junction between cells is critical.^[^ ^32^ ^]^ The plakoglobin and microtubules were enrichment at cell–cell junctions (Figures 4b,d, and e), suggesting that cell–cell junctions play a key role in the regulation of cells by soft substrates, promoting the formation of highly metastatic clusters. Based on these findings, we propose the following model. LNCaP cells are sensitive to the mechanical strength of the substrate via FAK and are mechanically conducted into cells by F‐actin. When cells are subjected to low tension provided by the substrate, they simultaneously modulate plakoglobin expression and EMT (via F‐actin, FAK and microtubules). Then, cells with intercellular connections form clusters via F‐actin, cytoskeletal tension, and myosin, and increases the expression of CD44 (Figure 4f).

The above results suggest that the heterogeneous responses to mechanical force of the PC3 and LNCaP cells may be due to differences in gene expression related to mechanical responses. Gene expression sequencing showed that PC3 cells had higher adhesion‐related gene expression, whereas LNCaP cells had higher expression of intercellular junction‐related genes (Figure S12, Supporting Information, Extended Data Table).

## 3 Conclusion

Previous studies showed that PC cell behavior is regulated by external environmental forces.^[^ ^13^ ^]^ Here, we focused on the heterogeneity of the response to mechanical force of PC cells isolated from different metastatic sites. PC cells from different metastatic sites have a heterogeneous response to external environmental mechanical force, and this heterogeneity produces two different patterns of metastasis. Regarding the metastasis of PC cells to bone, mechanically induced YAP/TAZ nuclear localization via high adhesion gene expression results in more efficient PC3 cell proliferation and migration in an extracellular environment characterized by high modulus. Regarding the metastasis of PC cells to the lymph, low adhesion and strong intercellular connections lead to the formation of LNCaP clusters under low modulus in the lymphatic system, and thus to efficient proliferation and metastasis.

The formation of CTC clusters plays an important role in cancer metastasis.^[^ ^25^, ^28^ ^]^ However, the mechanism underlying cluster formation is poorly understood. The current results reveal that the mechanical properties of the extracellular environment may play a role in the formation of CTC clusters.

These findings represent a major advance in our understanding of cancer metastasis. The response of metastatic cancer cells to the mechanical properties of the external environmental is heterogeneous; this heterogeneity is associated with an altered cancer cell gene expression, which ultimately results in different metastatic patterns that help cancer cells adapt to the mechanical properties of the metastasis site. This phenomenon has never been discussed in detail before, and it is important to fully understand the responses of cancer cells to diverse mechanical environments during cancer cell metastasis to facilitate diagnosis and treatment.

## 4 Experimental Section

##### Preparation of PDMS substrate

PDMS substrates of varying rigidity was prepared based on the previous literature.^[^ ^12^ ^]^ The SYLGARD components A and B (Sylgard 184; Dow Corning, Midland, MI, USA) were mixed at 50:1 and 100:1 ratios and spread on cell culture vessels (10‐ or 15‐cm‐diameter dish, 48‐ or 6‐well plate) or coverslips (13 mm), and cured at 70 °C overnight. Sulfo‐SANPAH solution (0.1 mg mL^−1^, 22 589; Thermo Scientific, Waltham, MA, USA) was dropped on the surface of the PDMS substrate, irradiated for 10 min with UV light, and irradiated again for 5 min after the removal of the sulfo‐SANPAH solution. The PDMS substrate was then coated with collagen I (25 µg mL^−1^) after washing twice with phosphate‐buffered saline (PBS). The mechanical properties of the PDMS substrates were characterized using a rotational rheometer (HAAKE MARS III; Thermo Scientific). Parallel plates were used in oscillation mode (0.1–10 Hz, 25 °C) and the results were analyzed using RheoWin Data Manager software (Thermo Scientific).

##### Cell lines

Four mycoplasma‐negative human PC cell lines (PC3, LNCaP, C4‐2B, and DU145), one mycoplasma‐negative human prostatic hyperplasia cell line (BPH‐1), and one mycoplasma‐negative human renal epithelial cell line (293) were obtained from Second Military Medical University (Shanghai, China). LNCaP, C4‐2B, DU145, and BPH‐1 cells were cultured in 1640 (R2405; Sigma, St. Louis, MO, USA) supplemented with 10% v/v fetal bovine serum (FBS; 10091148; Gibco, Grand Island, NY, USA), 100 U mL^−1^penicillin, and 100 µg mL^−1^ streptomycin (10378016; Gibco). PC3 cells were cultured in F12 (N6658; Sigma) supplemented with 10% v/v FBS, 100 U mL^−1^ penicillin, and 100 µg mL^−1^ streptomycin. 293 cells were cultured in Dulbecco's Modified Eagle Medium (DMEM) (D5796; Sigma) supplemented with 10% v/v FBS, 100 U mL^−1^ penicillin, and 100 µg mL^−1^ streptomycin. All cells were cultured at 5% CO_2_ and 37 °C.

##### Cell proliferation assays

Substrates with different mechanical properties were prepared in 48‐well plates. Then, the same number cells (1 × 10^5^) were cultured on the substrate for 12–48 h and cells proliferation were measured using EdU staining.^[^ ^33^ ^]^ Briefly, cells were incubated for 1 h in medium supplemented with 10 µM EdU (C10639; Invitrogen, Carlsbad, CA, USA), fixed with 4% paraformaldehyde for 15 min at room temperature, and permeabilized with 0.5% Triton X‐100 in PBS for 5 min. Then, 100 µL Click‐iT Plus reaction cocktail (C10639; Invitrogen) was added to each well and incubated in the dark for 30 min at room temperature. After washing three times with PBS, cells were incubated with 1 µg mL^−1^ Hoechst 33342 (62249; Thermo Scientific) for 15 min in the dark at room temperature. Finally, the Hoechst 33342 was removed, and the cells were washed three times with PBS and imaged with a fluorescence microscope (Axio Observer; Zeiss, Oberkochen, Germany). Image‐Pro Plus 6.0 software (Media Cybernetics, Silver Springs, MD, USA) was used for image analysis.

##### Cell migration assays

In vitro cell migration was measured using Transwell assays.^[^ ^34^ ^]^ Cells cultured on different substrates were collected separately. Then, cells resuspended in FBS‐free medium, and added (200 µL, 5 × 10^4^) to the culture insert (353097; Corning) and medium containing 5% FBS (800 µL) was added to the well and cells were cultured at 37 °C with 5% CO_2_. After 12 h of culture, the cells were fixed and stained with 0.1% crystal violet, wiped carefully to remove cells that did not pass through the membrane, and imaged. The crystal violet was eluted from cells with 33% acetic acid, and absorbance was measured at 570 nm to determine the number of migrated cells.

##### Subcutaneous tumors of nude mice

PC3 and LNCaP cells were cultured on different substrates. After 7 days of culture, one million cells were collected separately, resuspended in 50 µL 1640, and injected subcutaneously into the upper armpit of the 6‐week‐old male nude mice (BALB/c; purchased from Second Military Medical University; Accreditation number: SYXK 2017‐0004). Tumors were collected after 4 weeks and their diameters were measured.^[^ ^18^ ^]^

##### Tail vein injection

LNCaP cells were labeled with luciferase for tracking^[^ ^35^ ^]^ and cultured on different substrates. After 7 days of culture, 100 000 cells were collected separately, resuspended in 200 µL 1640, and injected into 6‐week‐old male nude mice (BALB/c, purchased from Second Military Medical University; Accreditation number: SYXK 2017‐0004) through the tail vein. Mice were imaged after 20 days (IVIS Lumina system; Xenogen, Alameda, CA, USA), with body weight changes being recorded over time. D‐Luciferin (88293; Thermo) was intraperitoneally injected into nude mice (150 mg kg^−1^) and they were imaged after 10 min. Then, the mice were sacrificed and their organs were harvested; paraffin sections were then stained with H&E to evaluate cell metastasis. All experiments have been approved by Animal Care and Ethics Committee of the Second Military Medical University.

##### Immunofluorescence imaging

Cells growing on different substrates were fixed with 4% paraformaldehyde for 10 min at room temperature and incubated with 0.1% Triton X‐100 in PBS for 3–5 min. After blocking with blocking solution containing 3% bovine serum albumin (BSA; A1933; Sigma) for 45 min, cells were incubated with antibodies (YAP1, H00010413‐M01, Novus, Littleton, CO, USA; pFAK, #8556; Myo‐II, #3403; Plakoglobin, #2930; *β*‐Tubulin, #2128; and CD44, #3570; all from Cell Signaling Technology, Danvers, MA, USA) overnight at 4 °C. Then, cells were incubated with secondary antibody (A32740 or A32742; Invitrogen) or phalloidin (O7466; Invitrogen) dissolved in PBS containing 1% BSA for 2 h. After washing three times with PBS for 5 min, cells were incubated with Hoechst 33342 (62249; Thermo Scientific) for 15 min at room temperature, washed again three times with PBS for 5 min, and imaged using a fluorescence microscope (Axio Observer; Zeiss) or confocal microscope (Ti‐E; Nikon, Tokyo, Japan).

For cross‐linked collagen substrates, cells were blocked with 5% BSA for 1 h, co‐incubated with primary and secondary antibodies against collagen I (ab6308; Abcam, Cambridge, UK), and analyzed using fluorescent immunoassays with the Axio Observer instrument.

##### Cell transfection

For YAP/TAZ knockdown via RNA interference,^[^ ^19^ ^]^ cells were transfected with an empty vector (pLKD‐CMV‐EGFP‐2A‐Puro‐U6‐shRNA [GL407], Obio Technology (Shanghai, China) and plasmids (pLKD‐CMV‐EGFP‐2A‐Puro‐U6‐shRNA [YAP/TAZ] GL407NC2; Obio) carrying interfering targets (YAP: GACAUCUUCUGGUCAGAGA dTdT; TAZ: ACGUUGACUUAGGAACUUU dTdT) using lentivirus. PC3 cells were plated in 24‐well plates, grown to 30–40% confluence, and incubated for 12 h after addition of lentivirus. Media was changed after 48 h, and cells were incubated in media containing puromycin (10 µg µL^−1^, A1113803; Gibco) for 5–7 days to select positive clones.

Cells were transfected with a luciferase‐expressing plasmid (pLenti‐CBh‐3FLAG‐luc2‐tCMV‐mNeonGreen‐F2A‐Puro, H7656; Obio) using lentivirus. LNCaP cells were plated onto 24‐well plates, grown to 30–40% confluence and incubated for 12 h after addition of lentivirus. Media was changed after 48 h, and cells were incubated in media containing puromycin (10 µg µL^−1^) for 5–7 days to select positive clones.

##### Western blotting

Cells were lysed with RIPA buffer (89900; Thermo Scientific) supplemented with protease and phosphatase inhibitors (A32959; Thermo Scientific) and proteins were quantified using bicinchoninic acid assays (23225; Thermo Scientific). Then, cell‐derived proteins were separated by SDS‐PAGE and transferred to polyvinylidene fluoride (PVDF) membranes (IPVH00010; Millipore, Burlington, MA, USA) by wet transfer. The membranes were blocked with 5% m/v BSA in tris‐buffered saline/tween (TBST) for 3 h and incubated with primary antibodies (TAZ, ab224239, Abcam; YAP1, H00010413‐M01, Novus, Littleton, CO, USA; FAK, #71433; Cell Signaling Technology; pFAK, #8556; Cell Signaling Technology; talin, #4021; Cell Signaling Technology; integrin‐b1, #34971; Cell Signaling Technology; GAPDH, #2118; Cell Signaling Technology; Vinculin, #13901; Cell Signaling Technology; CD44#3570; Cell Signaling Technology; plakoglobin, #2930; Cell Signaling Technology; vimentin, #5741; Cell Signaling Technology; E‐cadherin, #14472; Cell Signaling Technology; N‐cadherin, #13116; Cell Signaling Technology; b‐Tubulin, #2128; Cell Signaling Technology) overnight at 4 °C. They were then incubated with horseradish peroxidase (HRP)‐conjugated anti‐rabbit (Cell Signaling Technology; 7074S) or anti‐mouse (Cell Signaling Technology; 7076S) secondary antibodies for 1 h at room temperature after washing with 0.1% Tween 20 in PBS. The protein signals were developed using a chemiluminescence system (ChemiScope; Clinx Science Instruments Co. Ltd, Shanghai, China).

YAP1 dephosphorylation was identified using Phos‐Tag SDS‐PAGE. Briefly, 50 µM Phosbind acrylamide (F4002, ApexBio) and 100 µM MnCl _2_ were added to SDS‐PAGE to prepare Phos‐Tag SDS‐PAGE.^[^ ^24^ ^]^ The cell‐derived proteins were separated by Phos‐Tag SDS‐PAGE, and the gel was incubated in a transfer solution containing Ethylene Diamine Tetraacetic Acid (EDTA) (5 mM) for 20 min (three times). Then, the gel was incubated in transfer solution twice for 10 min each time and the proteins were transferred to PVDF membranes (IPVH00010; Millipore) by wet transfer. The membranes were blocked with 5% m/v BSA in TBST for 3 h and incubated with primary antibody (YAP1, H00010413‐M01; Novus) overnight at 4 °C followed by HRP‐conjugated anti‐mouse (7076S; Cell Signaling Technology) secondary antibody for 1 h at room temperature after washing with TBST. The protein signals were developed using the ChemiScope system.

##### H&E staining

Dewaxed mouse tissue sections were stained in hematoxylin for 30 min at room temperature, rinsed with tap water for 15 min, immersed in hydrochloric acid for 5 s, and rinsed again in tap water. The sections were then dehydrated and stained with 0.5% erythritol for 3 min at room temperature. They were then rinsed in 95% alcohol to wash away excess erythritol, dehydrated in absolute ethanol, placed in xylene, sealed with neutral gum, and imaged using a microscope (CX43; Olympus, Tokyo, Japan).

##### Inhibition assays

The F‐actin inhibitor cytoskeleton B (HY‐16928; MCE, Shanghai, China),^[^ ^23^ ^]^ microtubule inhibitor nocodazole (HY‐13520; MCE),^[^ ^19^ ^]^ FAK inhibitor PF‐573288 (HY‐10461; MCE),^[^ ^22^ ^]^ ROCK inhibitor Y‐27632 (HY‐10071),^[^ ^19^ ^]^ and Myo‐II inhibitor (‐)‐blebbistatin (HY‐13441; MCE)^[^ ^31^ ^]^ were added to cell culture medium (10 µM). PC3 cells were cultured on different substrates for 24 h and then co‐cultured with inhibitor‐containing medium for 3 h. LNCaP cells were cultured on different substrates for 3 days and then co‐cultured with inhibitor‐containing medium for 24 h.

##### Gene expression assays

PC3 and LNCaP cells were lysed with TRIzol (15596018; Invitrogen) and total RNA (*n* = 3) was extracted and quantified using the NanoDrop ND‐1000 instrument (ND‐1000; Thermo) and Agilent 2200 TapeStation system (Agilent, Santa Clara, CA, USA). Then, mRNAs from each sample were separated using polyA capture and sequenced by HiSeq (Illumina, San Diego, CA, USA). After sequencing, the raw data were filtered using Fastp software to remove low‐quality sequences, undetected sequences, and linker sequences, and named” Clean Data.” The Clean Data sequences were compared with the genomic sequences using the Hisat2, MapSplice, Star, and Tophat2 algorithms, and a bam file of the genomic alignment was obtained; the chromosome distribution and genomic alignment rate data were obtained from the “Sample.bam” file. Next, gene expression data were exported in a file named “Counts.txt data.” The genes in Counts.txt were screened with the EBSeq R package to identify differentially expressed genes (log2FC > 0.585 or < −0.585; < 0.05 was defined as significant); these data were included in a file named “Dif‐Gene.txt.” By reference to Gene Ontology (GO) databases, such as NCBI, UNIPROT, SWISSPROT, and AMIGO, a functional analysis of the differentially expressed genes in Dif‐Gene.txt was performed to obtain functional enrichment data for the differential genes, which were included in a file named “GO analysis.”

##### Data analysis

All data were analyzed using *t*‐tests in Matlab R2014a software (MathWorks, Natick, MA, USA). For fluorescence images, Image‐Pro Plus 6.0 (Media Cybernetics) was used for quantitative photometric analysis. All gene expression sequencing data were analyzed using Novel Bio cloud computing services (http://www.novelbio.com/yun.html).

## Conflict of Interest

The authors declare no conflict of interest.

## Supporting information

Supporting Information
