# Supplementary material for: Heterogeneous Responses to Mechanical Force of Prostate Cancer Cells Inducing Different Metastasis Patterns
Source: Adv Sci (Weinh). 2020 Jun 17;7(15):1903583. doi: 10.1002/advs.201903583 (PMC7404165; doi:10.1002/advs.201903583)
Supplement: Supplementary file 1 — Supporting Information [file ADVS-7-1903583-s001.pdf]

© 2020 Wiley-VCH GmbH

ADVANCED SCIENCE
Open Access

Supporting Information

for *Adv. Sci.*, DOI: 10.1002/advs.201903583

Heterogeneous Responses to Mechanical Force of Prostate Cancer Cells Inducing Different Metastasis Patterns

Zhixiao Liu, Liujun Wang, Huan Xu, Qiqige Du, Li Li, Ling Wang, En Song Zhang, Guosong Chen, and Yue Wang**

Copyright WILEY-VCH Verlag GmbH & Co. KGaA, 69469 Weinheim, Germany, 2018.

Supporting Information

Title: Heterogeneous responses to mechanical force of prostate cancer cells inducing different metastasis patterns

Zhixiao Liu, Liujun Wang, Huan Xu, Qiqige Du, Li Li, Ling Wang, En Song Zhang, Guosong Chen, Yue Wang **

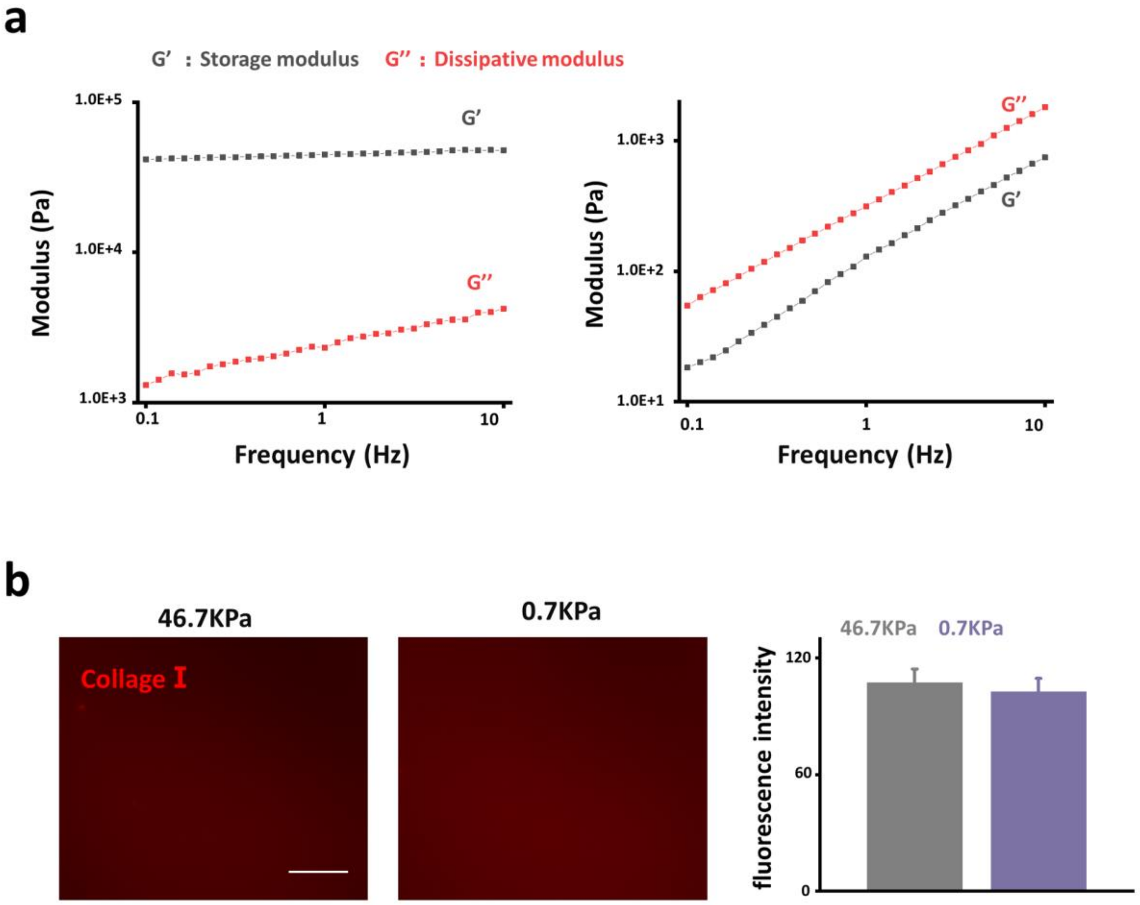

Figure S1. Characterization of polydimethylsiloxane (PDMS)-prepared substrates. **a**, A rotating rheometer was used to characterize the stiffness of the PDMS substrate (50:1, left; 100:1 right). Storage modulus measured at 0.1 Hz was used to represent the stiffness of the substrate (50:1, 46.7 KPa; 100:1, 0.7 KPa). **b**, Immunofluorescence imaging was used to quantify collagen on the substrate surface (*n* = 9; scale bar, 50 μm).

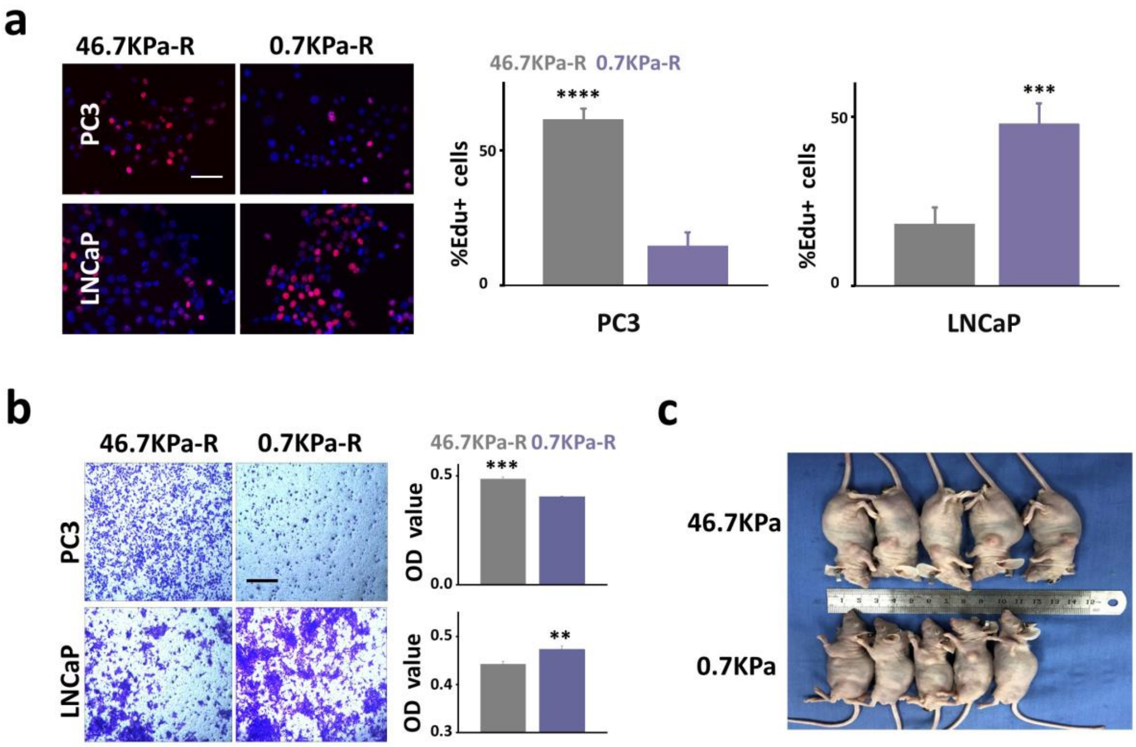

Figure S2. External environmental mechanical forces regulate cell behavior is irreversible. **a-b,** Mechanical properties irreversibly regulate cell proliferation and migration. We collected cells after 7 days in culture on different substrates and re-plated them in ordinary 24 or 96-well plates for 3 days. 46.7KPa-R referred to cells that have been re-plated in ordinary 24 or 96-well plates for 3 days after 7 days of cultivation on a stiff substrate, and 0.7KPa-R referred to cells that have been re-plated in ordinary 24 or 96-well plates for 3 days after 7 days of cultivation on a sofr substrate. EdU (96-well) and transwell crystal violet staining (24-well) were then used to analyze cell proliferation and migration. **a,** 46.7KPa-R PC3 has a higher proliferation rate than 0.7KPa-R PC3 cells (P=6.59397 × E-6). In contrast, 0.7KPa-R LNCaP has a higher proliferation (n = 3, P=2.675.2 × E-4, scale bar: 100 μm). **b,** Transwell crystal violet staining results (scale bar: 1 mm). 46.7KPa-R PC3 have a higher migration efficiency (P= 1.09027 × E-4), whereas 0.7KPa LNCaP cells have a higher migration efficiency (n = 3, P= 0.00332). **c,** *In vivo* tumorigenic ability of cells grown on different substrates. Using PC3 cells as an example, after 7 days of culture on substrates with different mechanical properties, cells were injected subcutaneously into nude mice to form tumors. Four weeks after injection, the mice were sacrificed and tumor diameters were measured. The results show that PC3 cells have greater tumorigenic ability after 7 days of growth on stiff substrates. *P<0.05; ** P<0.01; *** P<0.001; **** P<0.0001, t-tests.

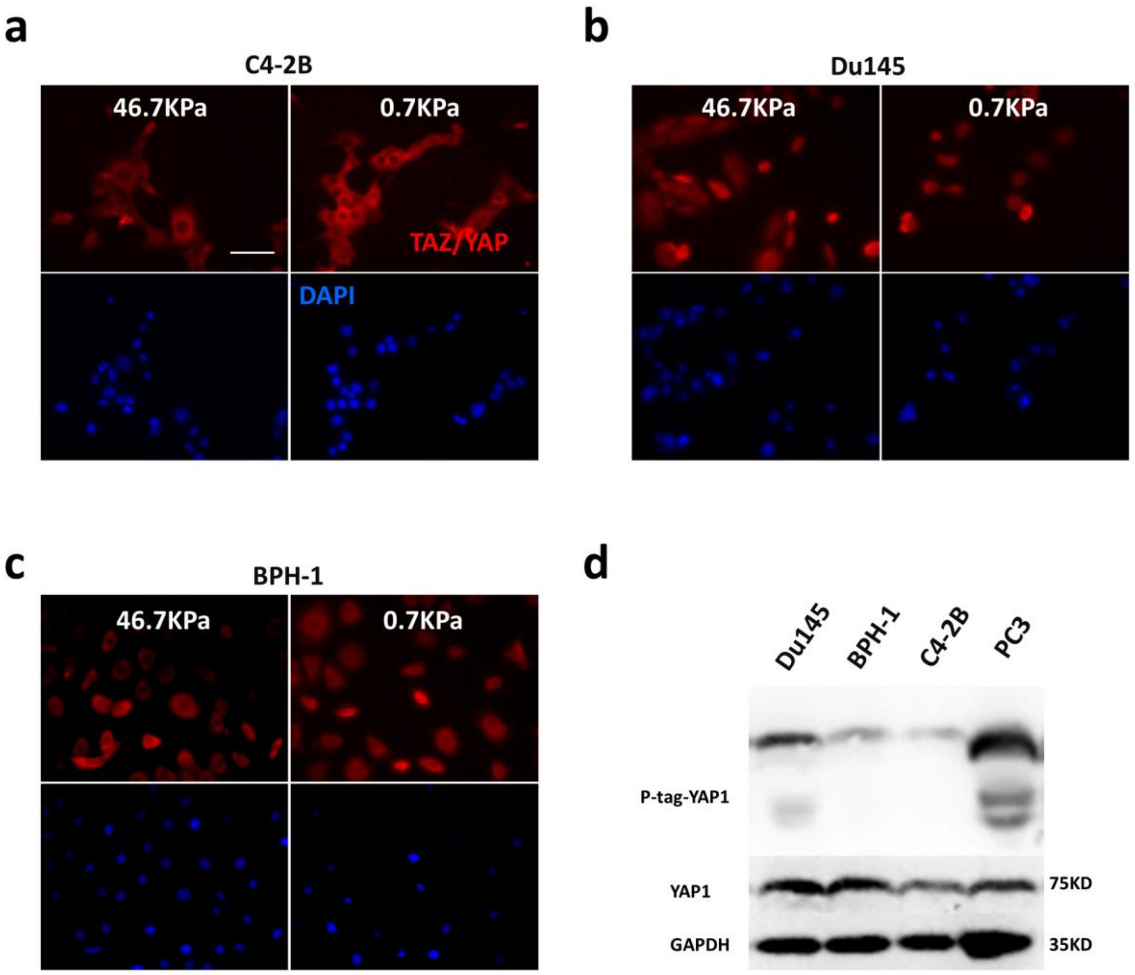

Figure S3. The mechanical properties of the substrate cause the lack of nuclear localization of YAP/TAZ seen in stiff substrate-insensitive prostate cancer cells. **a–c**, YAP/TAZ immunofluorescence imaging showing that the mechanical properties of the substrate prevent YAP/TAZ nuclear localization in C4-2B, Du145, and BPH-1 cells (scale bar, 50 µm). **d**, P-tag illustrating YAP1 dephosphorylation levels in PC3, C4-2B, DU145, and BPH-1 cells grown on stiff substrates. The results show that stiff substrates cannot induce YAP/TAZ dephosphorylation in C4-2B, DU145, and BPH-1 cells.

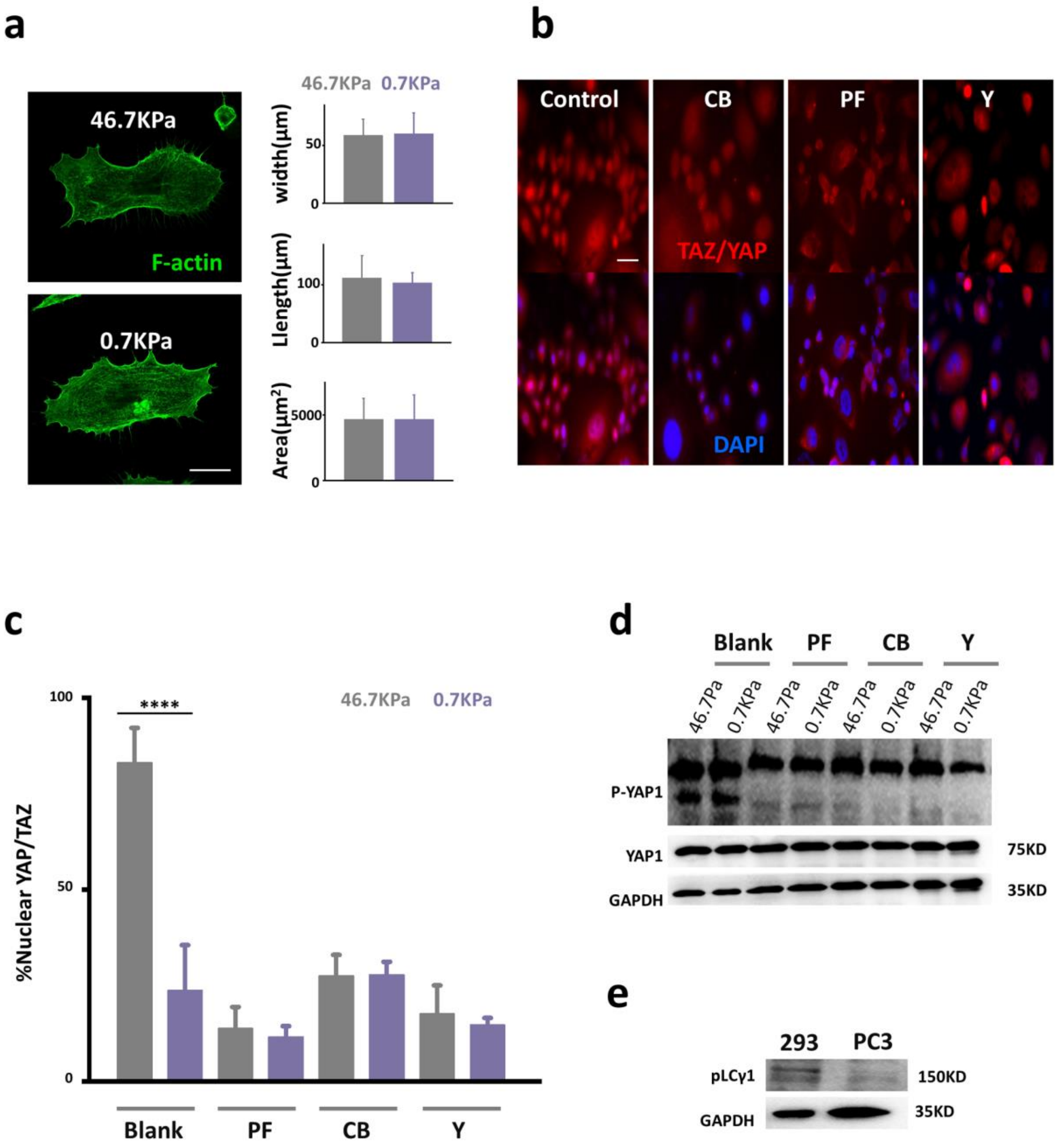

Figure S4. The mechanical properties of the substrate induce YAP/TAZ nuclear localization in PC3 cells. **a**, Morphology of PC3 cells grown on substrates with different mechanical properties. F-actin immunofluorescence images showing that there was no significant difference in the morphology of PC3 cells grown on different substrates (*n* = 100; scale bar, 10 μm). **b**, PC3 cells were plated on different substrates for 48 h and then incubated with inhibitors for 3 h. YAP/TAZ immunofluorescence images of PC3 cells grown on stiff substrates showing that the F-actin inhibitor CB, FAK inhibitor PF, and ROCK inhibitor Y inhibit stiff substrate-induced YAP/TAZ nuclear localization in PC3 cells. **c**, YAP/TAZ nuclear localization in PC3 cells grown on different substrates and incubated with CB, PF,

and Y for 3 h (scale bar, 50 μm). The results showed that, except for the blank control (*n* = 3, *P*=1.9975 × E-5), there was no significant difference in YAP/TAZ nuclear localization in PC3 cells grown on different substrates. **d**, P-tag demonstrating YAP1 dephosphorylation levels in PC3 cells grown on different substrates and incubated with inhibitors for 3 h. The inhibitors inhibit YAP/TAZ dephosphorylation in PC3 cells. **e,** pLCγ1 expression levels in PC3 cells and 293 cells. *$P<0.05$; ** $P<0.01$; *** $P<0.001$; **** $P<0.0001$, *t*-tests.

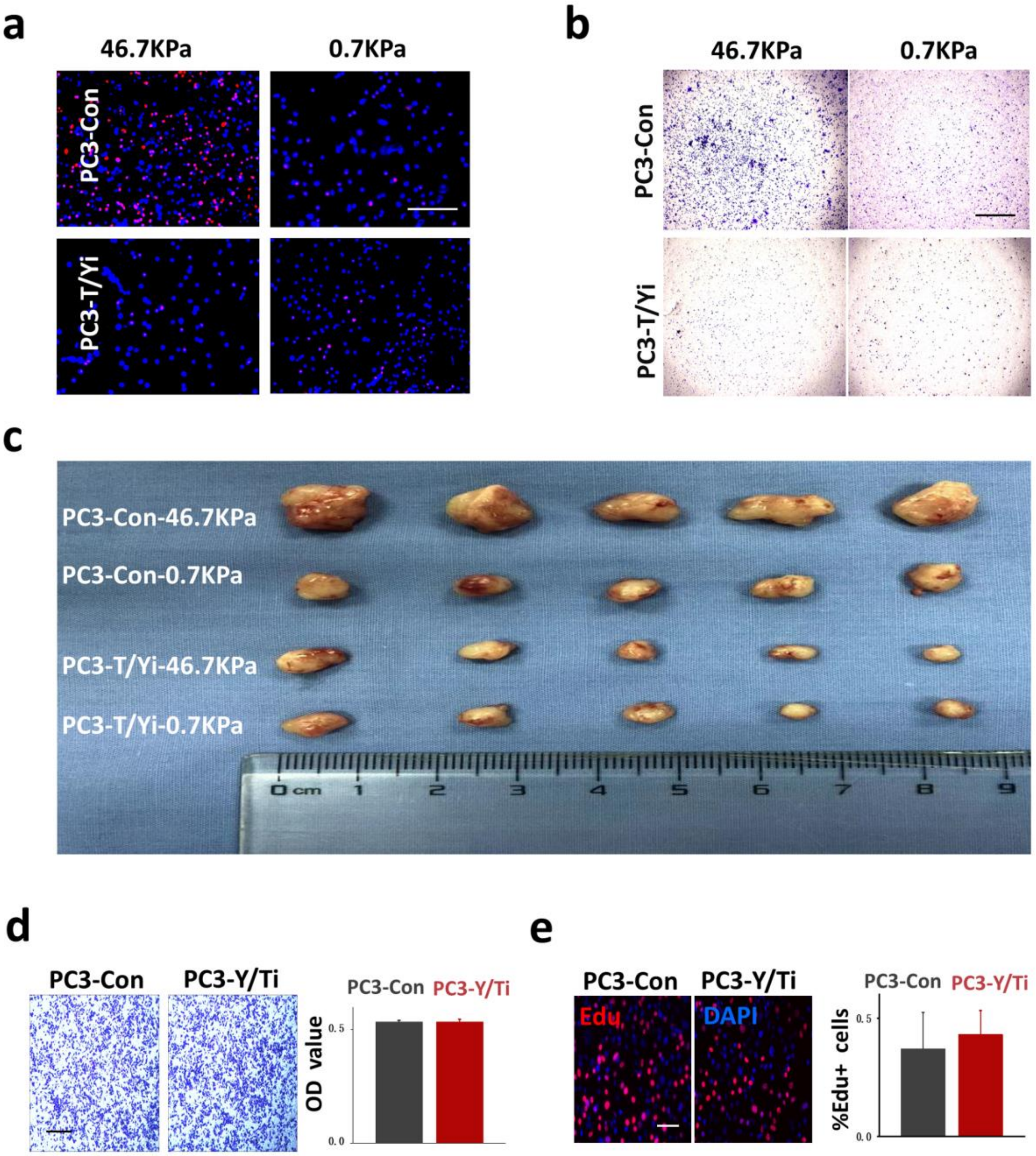

Figure S5. Knock down of YAP/TAZ expression via RNA interference inhibits the mechanical properties of PC3 cells grown on different substrates. **a**, PC3 cells were transfected with empty vector (PC3-Con) or YAP/TAZ-interfering RNA (PC3-T/Yi) and plated on different substrates; EdU staining was used to measure cell proliferation with fluorescence imaging after 48 h of growth (scale bar, 200 μm). **b**, PC3-Con and PC3-T/Yi cells were plated on different substrates and Transwell assays were used to measure cell migration after 4 days of growth (scale bar, 1 mm). **c**, PC3-Con and PC3-T/Yi cells were grown on different substrates. After 7 days of culture, cells were injected into the subcutaneous tumors of nude mice and imaged 4 weeks after injection. **d-e**, Knockout of the

YAP/TAZ gene has no significant effect on PC3 cells proliferation and migration on uncoated collagen culture dishes. PC3-Con and PC3-Y/Ti were plated on 24-well (Transwell) or 96-well (EdU) plates without collagen coating. After 48 hours of incubation, cell proliferation and migration were measured by EdU and Transwell. **d,** Transwell crystal violet staining results (scale bar: 1 mm).**e,** EdU staining results (scale bar, 100 μm).

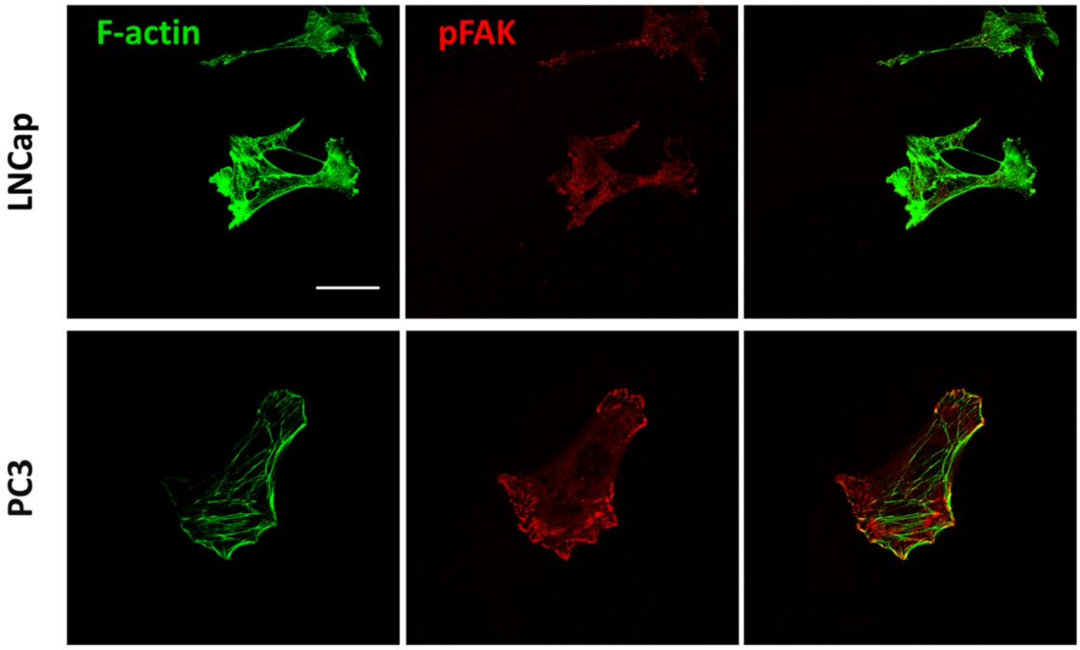

Figure S6. Immunofluorescence imaging of focal adhesion in PC3 cells and LNCaP cells. Green, F-actin; red, pFAK (scale bar, 25 μm).

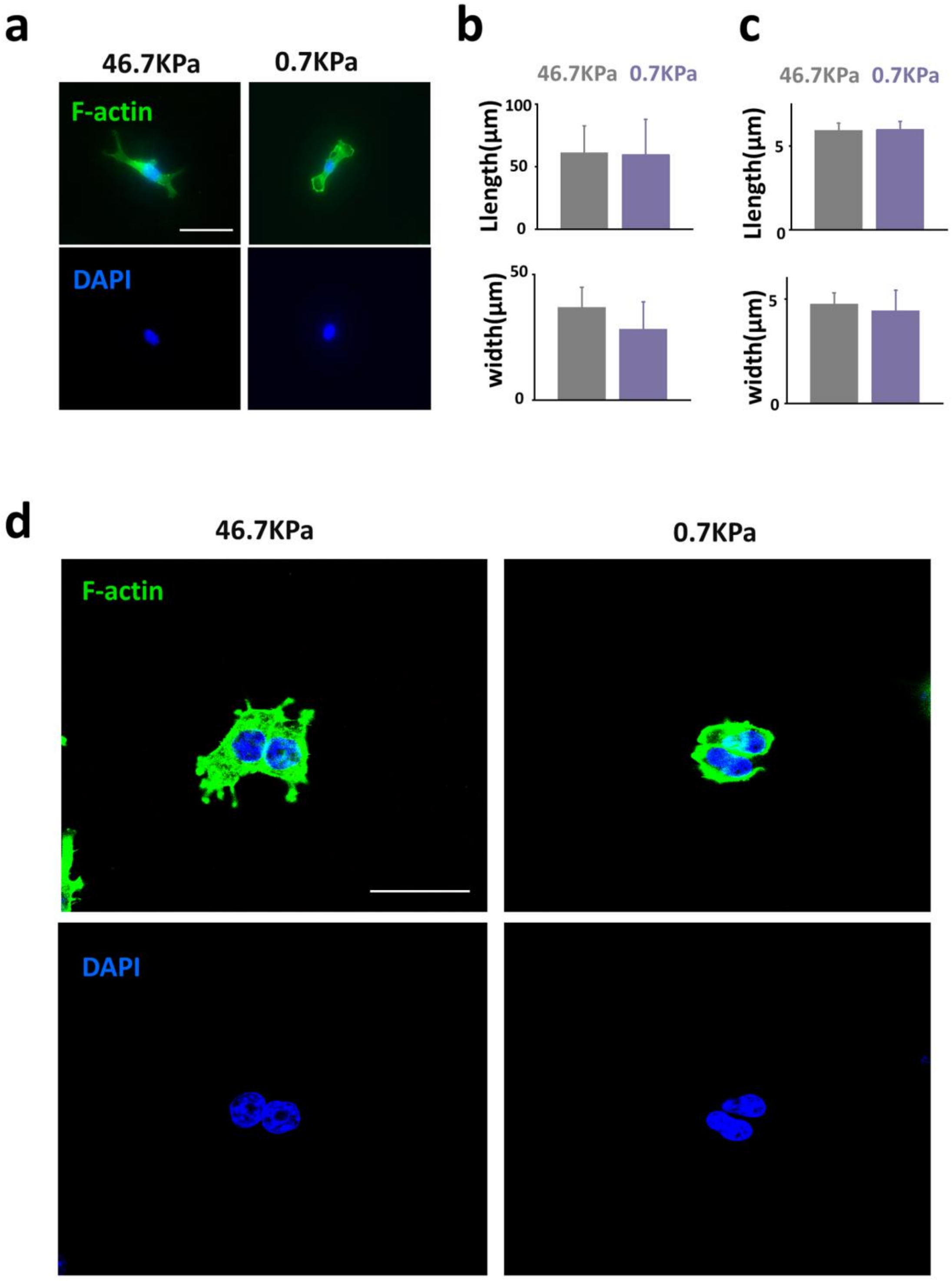

Figure S7. Soft substrate regulation of LNCaP cell behavior requires intercellular contact. **a**, F-actin immunofluorescence imaging of single LNCaP cells grown on different substrates without cell contact (scale bar, 10 μm, 4 days). **b**, Cell morphology was analyzed using fluorescent imaging and there were no significant differences in cell morphology between single LNCaP cells grown without cell contact on different substrates (*n* = 50). **c**, Nuclear

morphology was analyzed using fluorescent imaging and there were no significant differences in the morphology of nuclei from single LNCaP cells grown without cell contact on different substrates (*n* = 20). **d**, Immunofluorescence imaging of F-actin in LNCaP cells grown with intercellular contact; soft substrates regulate nuclear morphological changes in LNCaP cells grown with cell contact (scale bar, 10 μm, 4 days). *P<0.05; ** P<0.01; *** P<0.001; **** P<0.0001, *t*-tests.

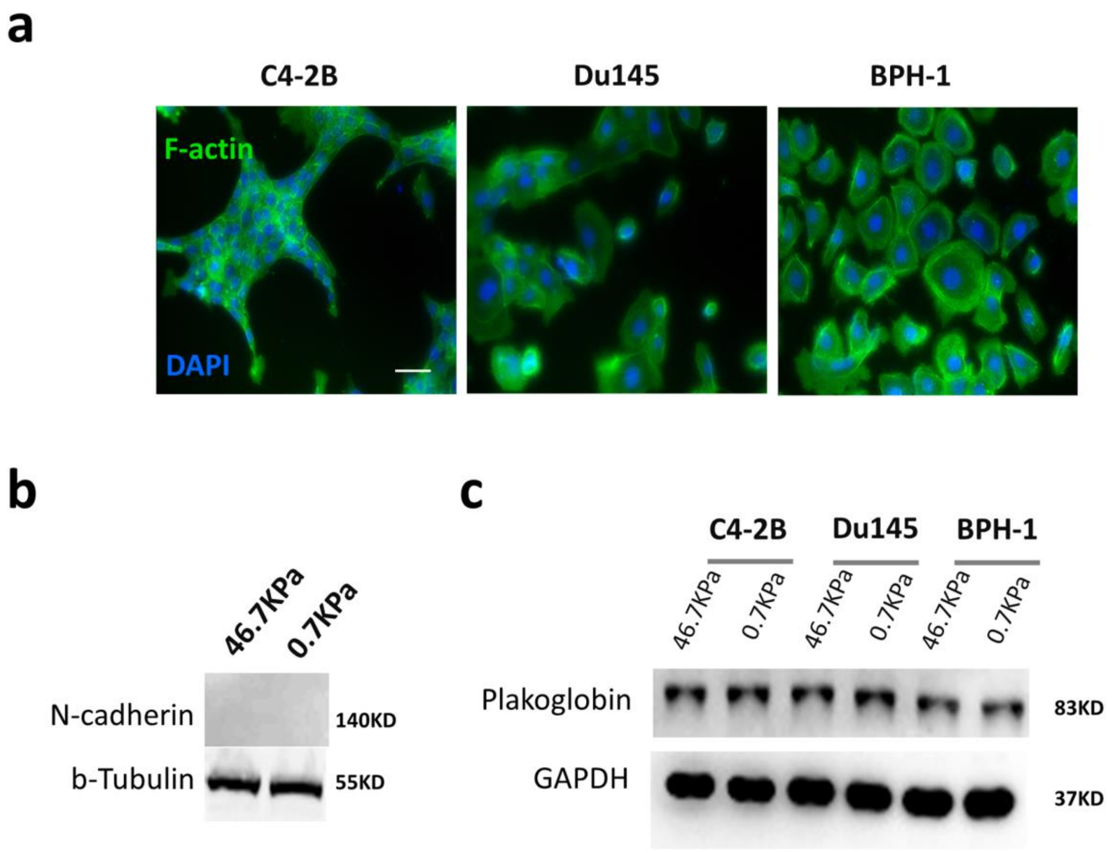

Figure S8. Soft substrates regulate cell clustering failure in soft substrate-insensitive cells. **a,** C4-2B, Du145, and BPH-1 cells that are insensitive to soft substrates did not form clusters on soft substrates after 4 days cultured. **b,** Different substrates do not affect N-cadherin expression in LNCaP cells. **c,** Soft substrates did not regulate plakoglobin expression, even in the presence of intercellular contact (scale bar, 50 μm, 4 days).

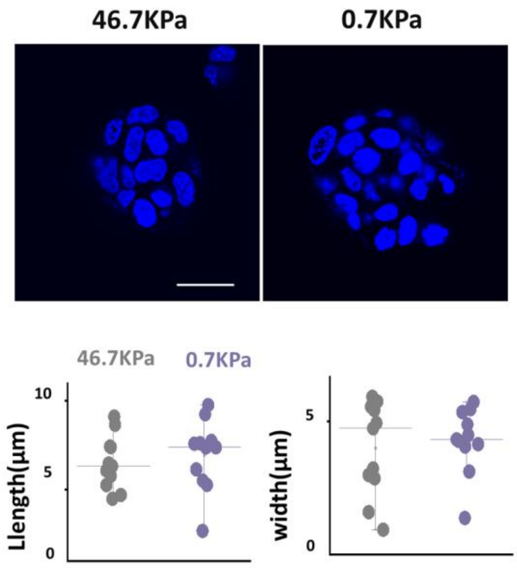

Figure S9. After the addition of a microtubule inhibitor (NO), LNCaP cells grown on hard substrates formed clusters similar to those seen on soft substrates, and the morphology of the nuclei changed ($n \geq 10$; scale bar, 10 μm, cultured on different substrates 3 days and co-cultivation with NO 24 h).

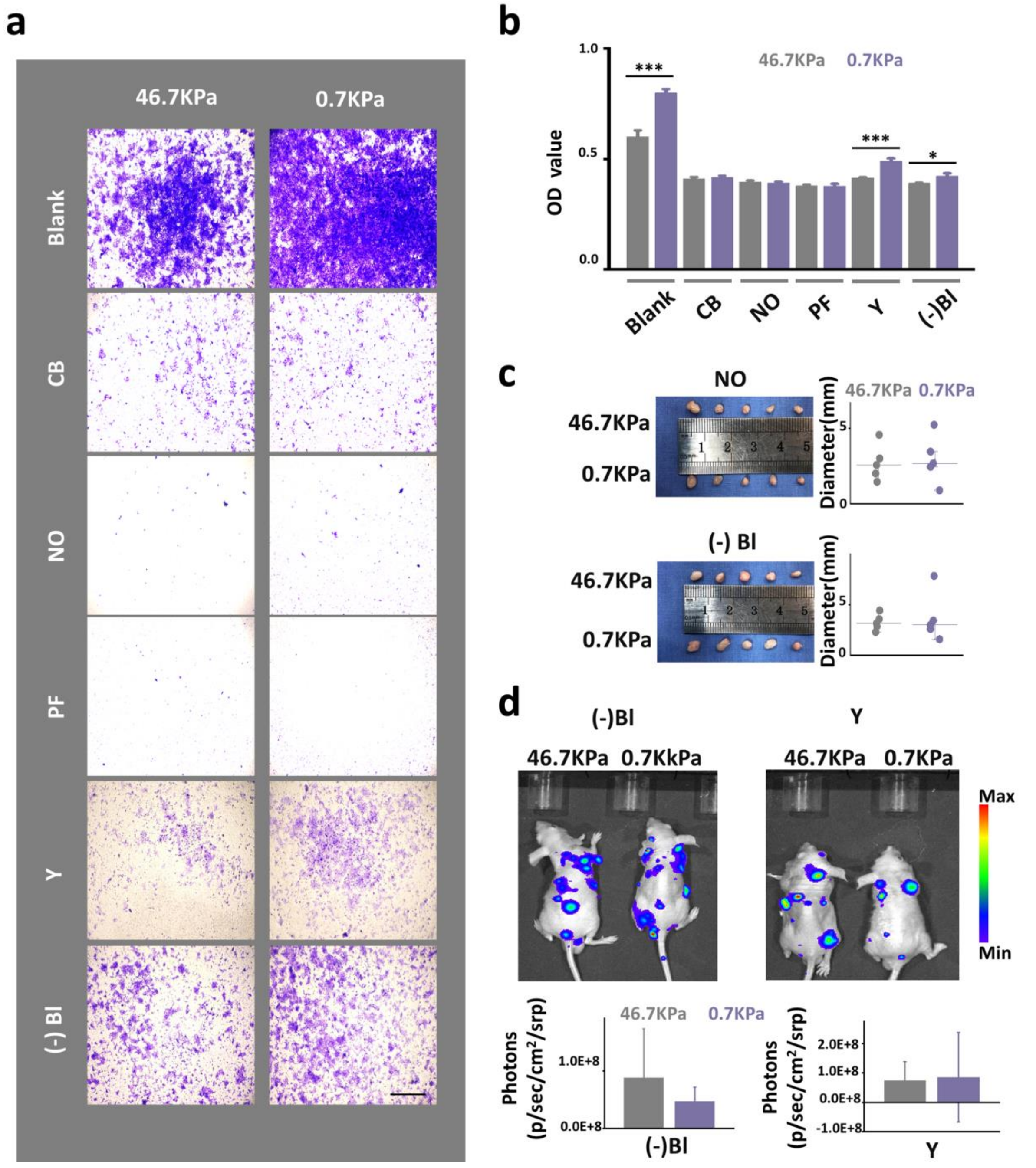

Figure S10. Inhibitors prevent soft substrates from promoting LNCaP cell migration. **a**, LNCaP cells were grown on different substrates for 48 h and incubated with inhibitors for 24 h; the cell migration ability was measured using Transwell assays (scale bar, 1 mm). **b**, Absorbance of crystal violet at 570 nm. The ROCK inhibitor Y and Myo-II inhibitor (-)Bl did not inhibit soft substrate-induced cell migration (n = 3; blank control, P=0.0005; Y, P=0.0005; (-)Bl, P=0.0142). **c**, Effects of different inhibitors on the tumorigenic ability of LNCaP cells grown on different substrates. LNCaP cells were treated with a microtubule

inhibitor NO or Myo-II inhibitor (-)Bl after 48 h of incubation on different substrates. After 5 days of culture, the cells were injected into the subcutaneous tumors of nude mice. There was no significant difference in the tumorigenic ability of LNCaP cells grown on different substrates after inhibitor treatment (n = 5). **d**, ROCK inhibitor Y and Myo-II inhibitor (-)Bl inhibit soft substrate-induced cell migration *in vivo*. Luciferase-labeled LNCaP cells were incubated on different substrates for 48 h and then treated with inhibitors for 5 days. The cells were then injected into nude mice via the tail vein. After 20 days, the mice were imaged *in vivo*. There was no significant difference in the *in vivo* migration ability of cells grown on different substrates and incubated with inhibitors. *P<0.05; ** P<0.01; *** P<0.001; **** P<0.0001, t-tests.

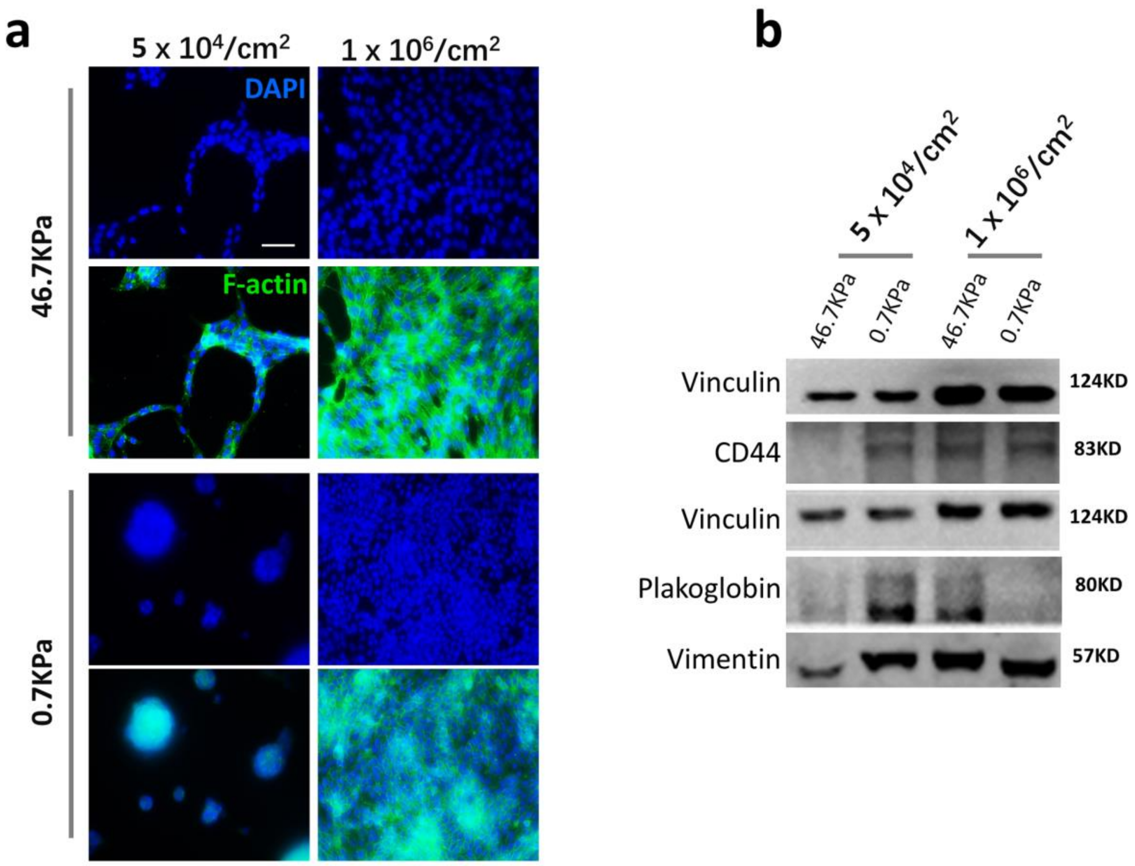

Figure S11. Effect of cell density on the LNCaP cell clustering induced by soft substrates. **a**, F-actin immunofluorescence imaging of LNCaP cells grown on different substrates, and at different cell densities (scale bar, 50 μm, 4 days). **b**, CD44, plakoglobin, and vimentin expression levels in LNCaP cells grown on different substrates at different cell densities after 4 days cultured.

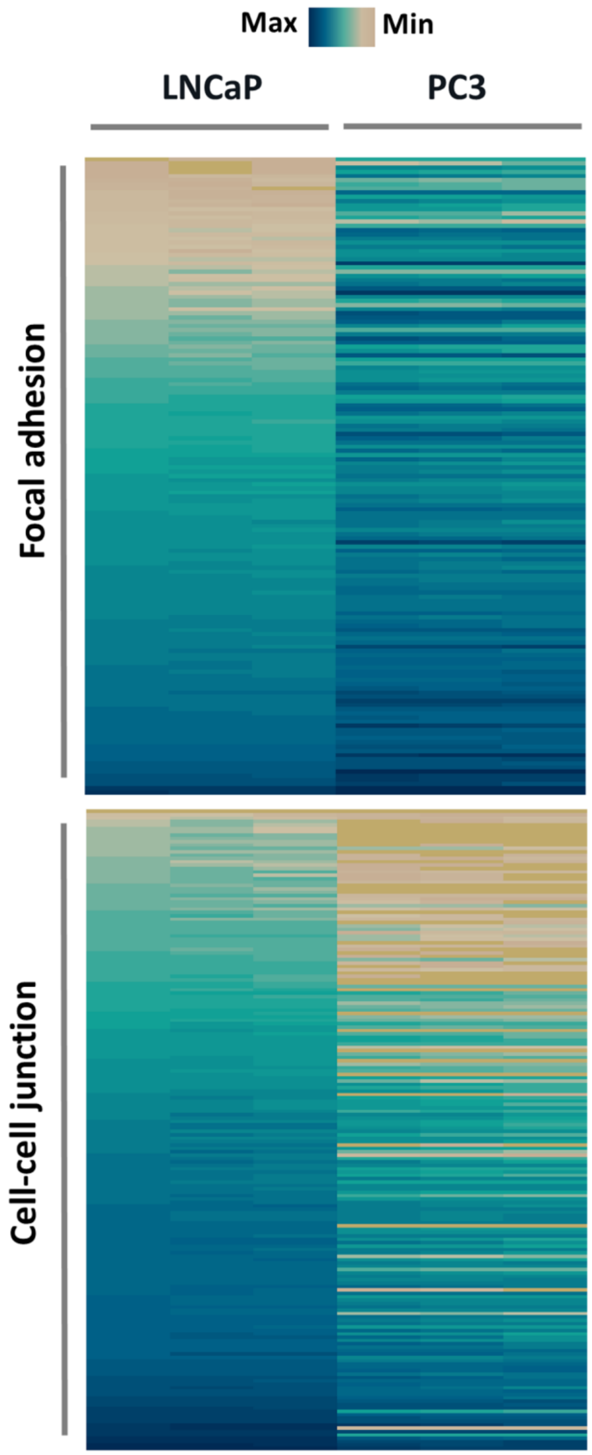

Figure S12. Heat map of focal adhesion-related and cell-cell junction-related protein expression in PC3 and LNCaP cells (n= 3).
